# Do Adolescents Like School-Based Mindfulness Training? Predictors of Mindfulness Practice and Responsiveness in the MYRIAD Trial

**DOI:** 10.1016/j.jaac.2023.02.016

**Published:** 2023-11

**Authors:** Jesus Montero-Marin, Verena Hinze, Catherine Crane, Nicola Dalrymple, Maria E.J. Kempnich, Liz Lord, Yasmijn Slaghekke, Kate Tudor, The MYRIAD Team, Saz Ahmed, Matt Allwood, Susan Ball, Marc Bennett, Sarah-Jayne Blakemore, Triona Casey, Katherine De Wilde, Darren Dunning, Eleanor-Rose Farley, Katie Fletcher, Lucy Foulkes, Poushali Ganguli, Cait Griffin, Kirsty Griffiths, Ben Jones, Nils Kappelmann, Konstantina Komninidou, Rachel Knight, Suzannah Laws, Jovita Leung, Emma Medlicott, Elizabeth Nuthall, Jenna Parker, Alice Phillips, Anam Raja, Lucy Palmer, Ariane Petit, Blanca Piera Pi-Sunyer, Isobel Pryor-Nitsch, Lucy Radley, J. Ashok Sakhardande, Jem Shackleford, Anna Sonley, Laura Taylor, Alice Tickell, Maris Vainre, Russell M. Viner, Brian Wainman, Lucy Warriner, Sarah Byford, Tim Dalgleish, Tamsin J. Ford, Mark T. Greenberg, Obioha C. Ukoumunne, J. Mark G. Williams, Willem Kuyken

**Affiliations:** a University of Oxford, Oxford, United Kingdom; b Teaching, Research & Innovation Unit, Parc Sanitari Sant Joan de Déu, Spain; c Consortium for Biomedical Research in Epidemiology & Public Health (CIBER Epidemiology and Public Health - CIBERESP), Spain; d University of Cambridge, Cambridge, United Kingdom; e Cambridgeshire and Peterborough NHS Foundation Trust, Cambridgeshire, United Kingdom; f Pennsylvania State University, Centre County, Pennsylvania; g University of Exeter, Exeter, United Kingdom

**Keywords:** school-based mindfulness training, adolescents, mindfulness practice, responsiveness, mixed methods

## Abstract

**Objective:**

We explored what predicts secondary school students’ mindfulness practice and responsiveness to universal school-based mindfulness training (SBMT), and how students experience SBMT.

**Method:**

A mixed-methods design was used. Participants were 4,232 students (11-13 years of age), in 43 UK secondary schools, who received universal SBMT (ie, “.b” program), within the MYRIAD trial (ISRCTN86619085). Following previous research, student, teacher, school, and implementation factors were evaluated as potential predictors of students’ out-of-school mindfulness practice and responsiveness (ie, interest in and attitudes toward SBMT), using mixed-effects linear regression. We explored pupils’ SBMT experiences using thematic content analysis of their answers to 2 free-response questions, 1 question focused on positive experiences and 1 question on difficulties/challenges.

**Results:**

Students reported practicing out-of-school mindfulness exercises on average once during the intervention (mean [SD] = 1.16 [1.07]; range, 0-5). Students’ average ratings of responsiveness were intermediate (mean [SD] = 4.72 [2.88]; range, 0-10). Girls reported more responsiveness. High risk of mental health problems was associated with lower responsiveness. Asian ethnicity and higher school-level economic deprivation were related to greater responsiveness. More SBMT sessions and better quality of delivery were associated with both greater mindfulness practice and responsiveness. In terms of students’ experiences of SBMT, the most frequent themes (60% of the minimally elaborated responses) were an increased awareness of bodily feelings/sensations and increased ability to regulate emotions.

**Conclusion:**

Most students did not engage with mindfulness practice. Although responsiveness to the SMBT was intermediate on average, there was substantial variation, with some youth rating it negatively and others rating it positively. Future SBMT developers should consider co-designing curricula with students, carefully assessing the student characteristics, aspects of the school environment, and implementation factors associated with mindfulness practice and responsiveness. SBMT teacher training is key, as more observed proficiency in SBMT teaching is associated with greater student mindfulness practice and responsiveness to SBMT.

If education is meant to prepare students for adult life, it must include social−emotional learning (SEL) in addition to academic attainment.^1^ SEL school programs aim to help students manage their emotional states, reach goals with empathy, maintain positive relationships, and make responsible decisions. Well-designed and implemented SEL programs have been found to improve students’ skills, attitudes, and social behavior.^2^ One approach to SEL that has shown promise is school-based mindfulness training (SBMT).^3^ Mindfulness training (MT) is an accessible and acceptable approach for adults with evidence for mental health promotion and positive effects on psychological distress, anxiety, depression, and well-being.^4^ MT involves teaching foundational skills, for example, emotional−behavioral self-regulation, which can enhance resilience and promote well-being in the face of stressors.

Because young people spend most of their time in schools, universal SBMT is a viable intervention to reach a broad range of students.^5^ However, implementing MT in schools requires careful consideration of the developmental stage of young people.^6^ A recent meta-analysis suggests that SBMT shows promise, but the evidence is inconclusive and calls for high-quality trials.^7^ In an adequately powered cluster randomized controlled trial (c-RCT) of universal SBMT vs SEL as usual (“My Resilience in Adolescence,” the MYRIAD trial), SBMT was found to support short-term changes in teacher burnout and school climate,^8^ with no evidence of effects on students’ mental health.^9^ In addition, this study raised the possibility that universal SBMT might be contraindicated for early adolescents and those at higher risk for mental health problems.^10^

Universal SBMT programs are likely to be effective only when students practice mindfulness skills and engage with the intervention.^11^^,^^12^ The degree to which a program stimulates the interest of participants, including their views regarding its perceived benefits, usefulness, enjoyability, and applicability to problems in daily life, is called “responsiveness,” and it is an important aspect of the implementation process.^13^^,^^14^ Alternative terms, previously used to refer to responsiveness, include “acceptability,” “receptiveness,” “satisfaction,” etc.^4^ A recent scoping review summarized the existing quantitative research on students’ experiences with SBMT and found that students reported low mindfulness practice outside of lessons and intermediate levels of responsiveness.^14^ There are studies in which students engage well with MT and respond more positively, but these tend to be with older students (eg, undergraduate) who have chosen to do the training.^15^ There were similarly mixed findings for the association between mindfulness practice and outcomes, and potential associations between responsiveness and outcomes.^14^ The authors highlighted the preliminary nature of the research on SBMT’s effectiveness, the processes through which SBMT might exert effects, and the potential influence of individual, teacher, school, and implementation factors on students’ mindfulness practice and responsiveness. Qualitative studies of students’ experiences with SBMT provide a mixed picture; some students have been enthusiastic, others negative, and others have expressed reservations about aspects of the curricula.^16^^,^^17^ Qualitative research has also highlighted the importance of high-quality teacher training to ensure that SBMT is delivered with fidelity,^18^ and the importance of a supportive school environment to ensure adequate implementation.^19^

Our study uses data from the largest trial of universal SBMT to date.^9^ It explores how secondary school students (11-13 years of age) engage with and respond to SBMT to better understand why the trial was not effective overall, and to generate hypotheses for future research. Hence, we sought to answer these questions: Do students in their first years of secondary school practice mindfulness exercises outside of lessons, when receiving SBMT? How do students respond to SBMT? Which student, teacher, school, and implementation factors are associated with students’ mindfulness practice and responsiveness? How do students experience SBMT?

## Method

This study is 1 of a series of studies exploring the effectiveness, cost-effectiveness, mechanisms, scalability, and implementation of SBMT. We used data from the MYRIAD trial, a c-RCT comparing SBMT with SEL as usual on students’ mental health (ISRCTN86619085; 03/06/2016). A mixed-methods approach was taken to help us make sense of the findings.^20^ We attended to both researchers’ framework and students’ reflections^21^ in elaborating, illustrating, and clarifying the results.^22^ The study was conducted and reported following mixed research recommendations.^23^

### Participants

We randomized 85 UK secondary schools (in England, Wales, Scotland, and Northern Ireland) to receive either SBMT (intervention) or to continue with existing SEL provision (control). Schools were eligible if they had an appointed permanent headteacher, had not been judged inadequate in their most recent official inspection, and had a strategy or structure in place for delivery of SEL curricula. Here, we report quantitative (experimental) and qualitative (phenomenological) data using those schools that were randomized to the SBMT trial arm (4,232 students in 43 schools [clusters]). Quantitative and qualitative phases occurred concurrently, drawing on the same sample that was representative of UK secondary schools and students,^24^ allowing both statistical generalization^9^ and an opportunity to explore and/or document diversity.^25^^,^^26^ At baseline, the mean (SD) age of students was 12.20 (0.60) years. Of the participants, 2,350 (56.5%) identified as female, and 3,237 (78.1%) identified as of White ethnicity.

### Procedure

Recruitment was carried out in 2 cohorts (cohort 1 [n = 551]: academic year 2016-2017; cohort 2 [n = 3,681]: 2017-2018). It involved consenting schools and teachers, providing parents with the opportunity to opt their children out (by returning a form to the teacher stating that they did not wish their child to participate), and assenting the young people. Students completed measures at baseline (0 months), pre-intervention (12 months), post-intervention (18 months), and 1-year follow-up (24 months). Lessons were delivered by schoolteachers as part of normal classroom teaching in addition to or in place of SEL (see Supplement 1, available online) over 1 school term in the second or third year of secondary school.

Teachers received personal MT over 8 weeks (ie, mindfulness-based cognitive therapy for life^27^), followed by a 4-day training course in how to deliver SBMT to students.^28^ The SBMT curriculum comprised a 10-session program, with sessions lasting 30 to 50 minutes each (“.b”; mindfulness in schools project^29^), that teaches mindfulness skills through a combination of psychoeducation, class discussion, and mindfulness practices. The program suggests out-of-school mindfulness practices at the end of each session, which are reviewed at the start of the next session. The SBMT, flow, and timeline can be found in Montero-Marin *et al.*^30^ The study design and procedures are presented in the trial protocol and update.^5^^,^^10^

The study was approved by the University of Oxford medical sciences division ethics committee (R45358/RE001; 23/05/2016), and was overseen by a data monitoring and ethics committee and the MYRIAD trial steering committee.

### Measures

Responses to closed- and open-ended questions were collected both online and in paper-and-pencil format. Full details of the measures are shown in Supplement 2, available online.

We assessed the frequency of students’ out-of-school mindfulness practice at post-intervention using a 6-item measure (eg, “During the course you were taught a range of mindfulness practices. How often did you practice being mindful?”). Questions were answered on a 6-point Likert scale (from 0 = never to 5 = every day). Responses were summed and divided by the number of items (range, 0-5). Student responsiveness to SBMT was evaluated at post-intervention using a 5-item measure that was adapted from a previous study.^31^ Questions (eg, “How much does what’s being taught in these lessons make sense to you in helping you to deal with issues young people face?”) were answered on an 11-point Likert scale (from 0 = not at all to 10 = a great deal). Responses were summed and divided by the number of items (range, 0-10).

Consistent with a conceptual model proposed in previous research,^14^^,^^30^ we assessed student, teacher, school, and SBMT implementation factors that might predict students’ mindfulness practice and responsiveness. Student variables included age, self-identified sex (male or female), ethnicity (White, Arab, Asian, Black/African/Caribbean, mixed/multiple ethnic groups, other ethnic groups), and risk for mental health problems (at risk, not at risk), derived by a latent profile analysis. Teacher variables included years of teaching experience, self-identified sex (male or female), ethnicity (White, other ethnic group), and burnout (Maslach Burnout Inventory−Educators Survey [MBI-ES]^32^ (range, 0-132). School variables were obtained largely from governmental online resources,^24^ and included urbanicity (urban, rural), the proportion of students within the school who were eligible for free school meals (ie, school-level economic deprivation), student-to-teacher ratio, and school SEL ethos (ie, the school values in relation to the way staff and students relate,^33^ using a bespoke index that ranges from 0 to 100). Implementation factors spanned pupil, class, and schools, and comprised dose (number of sessions students attended), fidelity (percentage of the SBMT curriculum that was covered), quality of delivery (using the mindfulness-based interventions teaching assessment criteria [MBI-TAC-Teach],^34^ ranging from 1 = incompetent to 6 = advanced), reach (percentage of students receiving >67% of the SBMT sessions out of the study’s year group school population^35^), and SEL delivery (whether SBMT replaced, was added, or was partially additive or partially substitutive of an existing SEL curriculum).

We asked about pupils’ experiences with SBMT at post-intervention using 2 open-ended questions focused on positive experiences and on difficulties or challenges.

### Data Analyses

Baseline student socio-demographic characteristics, student mindfulness practice and responsiveness, and teacher, school, and SBMT implementation factors were described using the mean (SD), median (interquartile range [IQR]), and frequency (percentage).

Mixed effects (multilevel) linear regressions were fitted using Stata v17.0 to estimate pupil-level, class-level, and school-level variance components for students’ mindfulness practice and responsiveness to SBMT, respectively. We reported the intra-cluster correlation coefficient (ICC) at the class and school levels, calculated as the proportion of the total variation in the outcomes that was attributable to those levels. We fitted unconditional means multilevel models with no fixed predictors to estimate the ICCs.

We explored whether student, teacher, school, and implementation factors accounted for variation in students’ mindfulness practice and responsiveness to SBMT at post-intervention. We examined the associations between each factor and students’ mindfulness practice and responsiveness, respectively, in univariable models. We allowed for correlations between observations nested within classes and schools by fitting 3-level mixed-effects linear regression models. Next, we estimated unique associations by fitting multivariable mixed-effects models, 1 model for each dependent variable (mindfulness practice, and student responsiveness), entering only those factors that provided *p* values <.1 in the univariable analyses. All models included design variables, namely, cohort status (cohort 1, cohort 2), UK country (England, Northern Ireland, Scotland, and Wales), school size (≥1,000 children, <1,000 children), and school sex (mixed, female participants only), as covariates.^5^

We quantified the effect size of the multivariable models using *R*^*2*^ at the individual, class, and school levels.^36^ We used 2-sided tests with a .05 significance level using complete case analyses and explored missing data. We controlled for the false discovery rate in our final multivariable models.^37^

To explore pupils’ experiences of SBMT, we extracted and merged the pupils’ answers to both open-ended questions. Two researchers independently performed thematic content analyses on the first half of the data using the constant comparative method,^38^ and developed *a posteriori* an empirical initial list of codes. Discrepancies were resolved through discussion, leading to an agreed-upon list of codes, which was then applied to the second half of the data. This second categorization was checked for discrepancies, which were resolved by a third party. Similar codes were grouped into underlying themes to represent a parsimonious solution. The final list of themes, using students wording where appropriate, was used to categorize a randomly selected 10% of entries by 2 researchers independently, obtaining a kappa value for concordance of 0.97 (95% CI = 0.96, 0.98). Finally, 1 researcher categorized all of the entries using the final list of themes. We present the themes, definitions, and verbatim examples, and have transformed them into frequencies and percentages, displaying a graphical representation of their endorsement (verification procedures are provided in Supplement 3, available online).

## Results

Baseline characteristics of participating students, teachers with MT, schools, and a description of the implementation factors are summarized in Table 1.

**Table 1:** Student, Teacher, School, and Implementation Factors of Study Sample

**Student characteristics (baseline)**	
Age, y, mean (SD)	12.2 (0.6)
Sex, female, n (%)	2,350 (56.5)
Ethnicity	
Arab, n (%)	80 (1.9)
Asian, n (%)	357 (8.6)
Black/African/Caribbean, n (%)	191 (4.6)
White, n (%)	3,237 (78.1)
Mixed/multiple ethnic groups, n (%)	183 (4.4)
Other ethnic groups, n (%)	97 (2.3)
Risk for mental health problems, high, n (%)	1176 (27.8)
**Teacher characteristics (baseline)**	
Years of experience, mean (SD)	14.1 (8.7)
Sex, female n (%)	122 (83.6)
Ethnicity, White, n (%)	137 (93.8)
Burnout, mean (SD), possible range 0-132	32.9 (14.9)
**School characteristics (baseline)**	
Urbanicity, urban, n (%)	36 (84)
Economic deprivation, % free school meals, mean (SD), possible range 0-100	13.2 (8.1)
Student−teacher ratio, mean (SD)	15.9 (1.7)
SEL ethos, mean (SD), possible range 0-100	50.0 (9.7)
**SBMT factors (post-intervention)**	
Fidelity, mean (SD), possible range 0-100	83.0 (12.1)
Dose, mean (SD), possible range 0-10	8.97 (2.1)
Quality, mean (SD), possible range 1-6	3.8 (0.8)
Reach, mean (SD), possible range 0-100	25.7 (11.4)
SEL delivery	
Additive, n (%)	82 (52.4)
Partially additive/partially substitutive, n (%)	30 (18.9)
Substitutive, n (%)	36 (23.1)
Not established, n (%)	9 (5.6)

Note: Age and risk for mental health were provided by 4,232 students. Sex was provided by 4,157 students. Ethnicity was provided by 4,145 students. Years of experience, sex, and ethnicity were provided by 146 teachers (a total of 156 teachers were recruited to deliver the student SBMT curriculum to the intervention arm). Burnout was provided by 121 teachers. Urbanicity, deprivation, student−teacher ratio, and social−emotional learning (SEL) ethos were provided for 43 schools. Fidelity was obtained from 164 classes. Dose was obtained from n = 3,265 students. Quality was obtained from 192 classes. Reach was obtained from 35 schools. SEL delivery (ie, additions to/replacement of existing SEL curriculum) was obtained from 157 classes. n (%): frequency (percentage). SBMT = school-based mindfulness training.

### Students’ Mindfulness Practice and Responsiveness to SBMT

Of the initial sample, a total of 3,613 students (85.4%) answered all items referring to mindfulness practice. Students with (vs without) mindfulness practice data identified more often as female participants, of White ethnicity, and at low risk for mental health problems (Table S1, available online). Students reported practicing only once on average (mean [SD] = 1.16 [1.07]; median [IQR] = 1.00 [0.17, 1.83]) (Table 2, Figure S1, available online). As we assessed multiple mindfulness exercises, the average student practiced once each, so the number of practices was more than once in total. Around 40% to 50% of students did not practice at all; 30% to 40% practiced each exercise from 1 time to 3 times, and 15% to 25% practiced each exercise at least once a week. A small proportion of the total variation in students’ mindfulness practice was at the class (ICC [95% CI] = 0.07 [0.04, 0.09]) and school (ICC [95% CI] = 0.01 [0.00, 0.03]) levels.

**Table 2:** Student Responsiveness and Home-Based Mindfulness Practice

Variables/items	N	Mean (SD)	Median (IQR)	0 n (%)	1 n (%)	2 n (%)	3 n (%)	4 n (%)	5 n (%)
**Mindfulness practice total score (range 0-5)**	3,613	1.16 (1.07)	1.00 (0.17 to 1.83)						
How often did you practice being mindful?	3,636	1.39 (1.35)	1.00 (0.00 to 2.00)	1,281 (35.2)	843 (23.2)	641 (17.6)	637 (17.5)	141 (3.9)	93 (2.6)
Pause and focus on breathing (ie, “.b: stop, breathe, and be”)	3,634	1.47 (1.37)	1.00 (0.00 to 2.00)	1,187 (32.7)	815 (22.4)	747 (20.6)	614 (16.9)	159 (4.4)	112 (3.1)
“Beditation” as a way of helping you get to sleep.	3,632	0.92 (1.29)	0.00 (0.00 to 1.00)	1,978 (54.5)	786 (21.6)	359 (9.9)	290 (8.0)	140 (3.9)	79 (2.2)
Be mindful in your everyday lives, for example walk a short distance mindfully, or eat a mouthful of food mindfully.	3,628	1.12 (1.32)	1.00 (0.00 to 2.00)	1,634 (45.0)	834 (23.0)	589 (16.2)	326 (9.0)	160 (4.4)	85 (2.3)
Notice stress in your body, eg, “stress signature” in difficult times, noticing where in the body you were feeling stress.	3,624	1.1 (1.28)	1.00 (0.00 to 2.00)	1,775 (49.0)	816 (22.5)	490 (13.5)	338 (9.3)	136 (3.8)	69 (1.9)
Think about your thoughts as passing objects such as buses, clouds, or rivers that pass through your mind.	3,628	1.4 (1.32)	1.00 (0.00 to 2.00)	1,812 (49.9)	741 (20.4)	521 (14.4)	322 (8.9)	144 (4.0)	88 (2.4)

	N	Mean (SD)	Median (IQR)	0 n (%)	1-2 n (%)	3-4 n (%)	5-6 n (%)	7-8 n (%)	9-10 n (%)
**Responsiveness total score (range 0-10)**	3,595	4.72 (2.88)	4.80 (2.60 to 7.00)						
How much did what was taught in these mindfulness lessons make sense to you in helping you to deal with issues young people face?	3,605	4.62 (3.06)	5.00 (2.00 to 7.00)	511 (14.2)	499 (13.9)	644 (16.2)	881 (24.7)	619 (17.2)	441 (12.2)
Do you think that these mindfulness lessons will help you have a healthier lifestyle?	3,606	4.23 (3.15)	4.00 (1.00 to 7.00)	666 (18.5)	562 (15.6)	672 (18.6)	784 (21.7)	484 (13.5)	438 (12.1)
Would you recommend these mindfulness lessons to a friend?	3,606	4.46 (3.28)	5.00 (1.00 to 7.00)	670 (18.6)	504 (14.0)	608 (16.9)	773 (21.4)	529 (14.7)	522 (14.5)
How important do you think it is that we make these lessons available to young people?	3,606	5.45 (3.27)	5.00 (3.00 to 8.00)	436 (12.1)	371 (10.3)	479 (13.2)	817 (22.6)	690 (19.1)	813 (22.5)
How successful do you believe these mindfulness lessons would be in decreasing problems or issues that young people have?	3,609	4.85 (3.08)	5.00 (2.00 to 7.00)	491 (13.6)	445 (12.3)	585 (16.2)	917 (25.4)	676 (18.7)	495 (13.8)

Note: Mindfulness practice presented the following value: 0 = never; 1 = once; 2 = 2 or 3 times; 3 = about once a week; 4 = several times a week; 5 = almost every day. Responsiveness ranged from 0 = not at all to 10 = a great deal, with no intermediate labels for the values in between. n (%): frequency (percentage). Beditation = practicing meditation in bed; IQR = interquartile range.

Of the initial sample, 3,595 students (85.0%) answered all items referring to responsiveness. Students with (vs without) responsiveness data identified more often as female participants, of White ethnicity, and at low risk for mental health problems (Table S1, available online). Responsiveness was in the middle of the scale (M [SD] = 4.72 [2.88]; median [IQR] = 4.80 [2.60, 7.00]) (Table 2, Figure S2, available online). Around half of the students rated SBMT as positive (5-6 or above), and half of the students thought that it was not very helpful. A small proportion of the variation in students’ responsiveness was at the class (ICC [95% CI] = 0.07 [0.04, 0.10]), and school (ICC [95% CI] = 0.04 [0.01, 0.06]) levels. The correlation between mindfulness practice and responsiveness was *r* = 0.49 (*p* < .001).

### Predictors of Students’ Mindfulness Practice

Table 3 displays the associations between the potential predictors and students’ mindfulness practice (ethnic minorities are described in Table S2, available online). In the multivariable model, after controlling for multiple testing, higher SBMT dose (regression coefficient [B] = 0.06; 95% CI = 0.03, 0.09) and higher SBMT quality (B = 0.16; 95% CI = 0.08, 0.25) were significantly related to higher students’ mindfulness practice. This model accounted for 2.7%, 15.0%, and 21.6%, of the variation in students’ mindfulness practice at the individual, class, and school levels, respectively.

**Table 3:** Regression Coefficients (B) for Predictors of Students’ Mindfulness Practice

	Univariable analysis			Multivariable analysis			B-H *p*
	B	95% CI	*p*	B	95% CI	*p*	
**Student**							
Age	–0.06	–0.12 to 0.01	.070	0.00	–0.07 to 0.08	**.**930	
Sex	0.09	0.03 to 0.15	.007	0.09	0.01 to 0.17	**.**020	
Ethnicity							
Arab	–0.19	–0.45 to 0.07	.161	–0.07	–0.36 to 0.23	.666	
Asian	0.14	0.01 to 0.27	.032	0.09	–0.06 to 0.24	.219	
Black/African/Caribbean	–0.08	–0.25 to 0.10	.405	–0.09	–0.29 to 0.12	.404	
White		Reference					
Mixed/multiple ethnic groups	–0.14	–0.31 to 0.03	.108	–0.13	–0.32 to 0.06	.186	
Other ethnic groups	0.28	0.04 to 0.51	.021	0.25	–0.02 to 0.51	.065	
Risk for mental health problems, high^a^	0.02	–0.06 to 0.10	.648				
**Teacher**							
Experience	–0.01	–0.01 to 0.00	.192				
Sex	0.18	0.01 to 0.35	.040	0.13	–0.05 to 0.31	.167	
Ethnicity, White^b^	0.06	–0.16 to 0.28	.607				
Burnout	0.00	–0.01 to 0.00	.484				
**School**							
Urbanicity	0.10	–0.09 to 0.29	.299				
Deprivation	0.01	0.00 to 0.02	.207				
Student/teacher ratio	–0.01	–0.05 to 0.04	.791				
SEL ethos	–0.01	–0.01 to 0.00	.091	–0.01	–0.02 to 0.00	.119	
**SBMT**							
Fidelity	0.00	0.00 to 0.01	.582				
Dose	0.06	0.03 to 0.08	<.001	0.06	0.03 to 0.09	<.001	∗∗∗
Quality	0.12	0.05 to 0.18	.001	0.16	0.08 to 0.25	<.001	∗∗∗
Reach	0.00	–0.01 to 0.01	.948				
SEL delivery							
Additive		Reference					
Partially additive/substitutive	–0.25	–0.40 to –0.09	.002	–0.16	–0.36 to 0.03	.102	
Substitutive	–0.12	–0.26 to 0.03	.117	–0.08	–0.27 to 0.11	.392	
Not established	–0.35	–0.60 to –0.09	.008	–0.30	–0.69 to 0.09	.130	

Note: Predictors with p < .1 in the univariable analysis were included in multivariable models. B-H p (Benjamini−Hochberg) adjusted p values to control for false discovery rate from multiple testing. Ref = category of reference; SEL = social−emotional learning; SEL delivery = additions to/replacement of existing SEL curriculum; SBMT = school-based mindfulness training (implementation factors).

a Category of reference: low risk for mental health problems.

b Category of reference: other ethnic groups. ∗*p* < .05; ∗∗*p* < .01; ∗∗∗*p* < .001.

### Predictors of Students’ Responsiveness to SBMT

Table 4 displays the associations between the potential predictors and students’ responsiveness to SBMT. In the multivariable model, after controlling for multiple testing, female participants (B = 0.42; 95% CI = 0.22, 0.62), students identifying as Asian (vs White) (B = 0.63; 95% CI = 0.23, 1.03; ethnic minorities are described in Table S2, available online), lower risk for mental health problems (B = 0.75; 95% CI = 0.51, 1.00), higher school-level economic deprivation (B = 0.04; 95% CI = 0.01, 0.07), higher SBMT dose (B = 0.16; 95%CI = 0.08, 0.23), and higher SBMT quality (B = 0.38; 95% CI = 0.16, 0.61), significantly predicted higher students’ responsiveness. This model accounted for 6.7%, 28.9%, and 47.8%, of the variation in students’ responsiveness at the individual, class, and school levels, respectively.

**Table 4:** Regression Coefficients (B) for Predictors of Students’ Responsiveness

	Univariable analysis			Multivariable analysis			B-H *p*
	B	95% CI	*p*	B	95% CI	*p*	
**Student**							
Age	–0.06	–0.24 to 0.12	.502				
Sex	0.37	0.20 to 0.54	<.001	0.42	0.22 to 0.62	<.001	∗∗∗
Ethnicity							
Arab	–0.27	–0.96 to 0.43	.455	0.31	–0.49 to 1.10	.449	
Asian	0.65	0.30 to 1.00	<.001	0.63	0.23 to 1.03	.002	∗∗
Black/African/Caribbean	0.19	–0.29 to 0.67	.439	0.19	–0.34 to 0.72	.480	
White		Reference					
Mixed/multiple ethnic groups	–0.13	–0.58 to 0.33	.587	–0.02	–0.52 to 0.48	.936	
Other ethnic groups	0.57	–0.06 to 1.21	.074	0.43	–0.26 to 1.12	.223	
Risk for mental health problems, high^a^	0.79	0.58 to 1.00	<.001	0.75	0.51 to 1.00	<.001	∗∗∗
**Teacher**							
Experience	0.01	–0.01 to 0.03	.403				
Sex	0.46	–0.02 to 0.95	.061	0.33	–0.16 to 0.82	.189	
Ethnicity, White^b^	0.06	–0.55 to 0.67	.853				
Burnout	–0.01	–0.02 to 0.01	.331				
**School**							
Urbanicity	0.40	–0.22 to 1.03	.207				
Deprivation	0.04	0.01 to 0.07	.008	0.04	0.01 to 0.07	.012	∗
Student/teacher ratio	0.01	–0.14 to 0.16	.911				
SEL ethos	–0.03	–0.05 to –0.01	.011	–0.02	–0.05 to 0.00	.098	
**SBMT**							
Fidelity	0.00	–0.01 to 0.02	.583				
Dose	0.17	0.10 to 0.24	<.001	0.16	0.08 to 0.23	<.001	∗∗∗
Quality	0.23	0.05 to 0.42	.014	0.38	0.16 to 0.61	.001	∗∗
Reach	–0.01	–0.03 to 0.02	.545				
SEL delivery							
Additive		Reference					
Partially additive/substitutive	–0.73	–1.21 to –0.25	.003	–0.60	–1.14 to –0.07	.028	
Substitutive	–0.45	–0.90 to –0.01	.048	–0.23	–0.74 to 0.28	.377	
Not established	–0.60	–1.35 to 0.16	.121	0.07	–0.98 to 1.12	.894	

Note: Predictors with p < .1 in the univariable analysis were included in multivariable models. B-H p (Benjamini–Hochberg) adjusted p values to control for false discovery rate from multiple testing. SBMT = school-based mindfulness training (implementation factors); SEL = social–emotional learning; SEL delivery (ie, additions to/replacement of existing SEL curriculum).

a Category of reference: low risk for mental health problems.

b Category of reference: other ethnic groups. ∗*p* < .05; ∗∗*p* < .01; ∗∗∗*p* < .001.

### Students’ Experiences With SBMT

A total of 3,191 students (75.4%) answered the open-ended questions regarding SBMT experiences, but only 1,329 (31.4%) provided minimally elaborated responses (ie, including more than just no, yes, some, etc). Table 5 presents the themes resulting from the content analysis of the elaborated answers, their definitions, examples, frequencies, and percentages (see Supplement 4 and Table S3, available online, for concordance details), and Figure S3, available online, shows their representation in a word cloud. Themes were endorsed in 1,905 categorizations (ie, some students mentioned more than 1 type of experience). Themes with a positive meaning included an increased awareness of feelings or sensations in one’s body (39.7% of the minimally elaborated responses), an increased perception of one’s ability to regulate one’s emotions (19.9%), gaining a new perspective on things (7.0%), gaining a new positive outlook in life (5.5%), improvements in focus (5.2%), curiosity (2.3%), appreciation (2.2%), self-confidence (1.7%), attentiveness to others (1.6%), better sleep (1.5%), and more vitality (1.0%). Themes with a negative meaning included an increase in weariness (10.3%), anxiety, stress, or worry (9.5%), boredom (7.2%), challenging thoughts (6.3%), feeling incompetent (6.3%), doubts about the effects of the practice (6.1%), not finding mindfulness useful (4.7%), focus deteriorations (3.9%; in general terms/at school: 0.7%; during the mindfulness task in particular: 3.2%), did not do it (2.0%), and the feeling that they had no choice (0.2%).

**Table 5:** Themes That Resulted From the Analysis of Students’ Experiences With the School-Based Mindfulness Training

Codes	Definitions/Examples^a^	n	% Relative^b^	% Total^b^
Physical sensations	An increased awareness of feelings or sensations in one’s body	527	39.7	16.5
	“Tingling feelings throughout my body”			
Managing feelings	An increased perception of one’s ability to regulate one’s emotions	264	19.9	8.3
	“They helped me in my everyday life when I got stressed by making it easier to calm down”			
Changes in energy	Changes in energy (both feeling more vitality and less dreariness, or on the contrary an increase in weariness or desire to sleep)	150	11.3	4.7
	“Felt like I had a lot of energy”; “It made me feel quite tired and sleepy”			
	-More vitality	13	1.0	0.4
	-Increase in weariness	137	10.3	4.3
Changes in focus	Changes in concentration, and/or consciousness of an experience, subject, or environment	121	9.1	3.8
	“It helped me focus on my year 8 exams”; “This made it hard to concentrate on the rest of my lessons”; “I sometimes found it hard to concentrate on what we were meant to be doing”			
	-Improvements	69	5.2	2.2
	-Deteriorations	52	3.9	1.6
	In general terms/at school	10	0.7	0.3
	During the mindfulness tasks	42	3.2	1.3
Distress	Feeling restless, anxious, uneasy, stressed, unpleasant, or worried	126	9.5	4.0
	“I got quite anxious after the ‘stress control' one; it made my anxiety worse during that lesson and when I was walking home”			
Boredom	Feeling disengaged because something is not interesting or there is nothing to do	96	7.2	3.0
	“The only part of these lessons that had any kind of effect on me, that was to experience the sheer boredom these classes produced”			
New perspective	Gain a new perspective on things	93	7.0	2.9
	“Some exercises made me think different in some situations”			
Rumination	An increase in challenging thoughts	83	6.3	2.6
	“I did a few lessons of it; some of the bad thoughts just kept coming back and I didn’t know what to do; this was challenging seeing as I don’t like what the thoughts were showing me”			
Can’t do it	Felt unable to practice mindfulness due to perceived incompetence	83	6.3	2.6
	“I can’t meditate for a long period of time”			
I don’t know	Not sure/cannot remember/did not understand the practice	81	6.1	2.5
	“I’m not sure”; “I can’t remember”; “I don’t know”			
Optimism	Positive outlook and happiness in life	73	5.5	2.3
	“When I participated in the lessons, I felt more positive and more optimistic about the future”			
Not useful	Didn’t find it useful and/or didn’t learn anything new	62	4.7	1.9
	“The biggest waste of time ever”			
Curiosity	Felt interested, curious, engaged, something to be learnt	30	2.3	0.9
	“I think that the dot-b lessons were very interesting, and I liked them a lot”			
Appreciation	An increase in appreciation by realizing things that you previously took for granted	29	2.2	0.9
	“It made me think more about the things I take for granted”			
Didn’t do it	Did not carry out or engage with the mindfulness practice outside of the lessons	26	2.0	0.8
	“I never done it after the lesson”			
Self-confidence	An increased feeling of trust in one’s ability and a positive self-image	23	1.7	0.7
	“I was a bit more confident in myself”			
Attentive to others	An increase in thoughtfulness and attention toward others	21	1.6	0.7
	“I noticed that I started to think more about the people in my life”			
Better sleep	An improvement in sleep quality	20	1.5	0.6
	“Sleeping was better”			
No choice	Didn’t feel they had a choice in whether to engage with the practice or that it was voluntary or optional	2	0.2	0.1
	“Miss__was teaching us and telling us to act a certain way when we get certain feelings, and it made me believe that it was forced on me rather than letting me have a choice in the matter of if I want to or if I don't want to do that”			*A*

Note: ^a^Examples are examples of student responses.

b % Relative uses as denominator the n = 1,329 students with a minimally elaborated response. % Total uses as denominator the n = 3,191 students who responded with something to the open-ended questions.

## Discussion

We found that most students did not engage in regular out-of-school mindfulness practice, with almost half of students reporting that they did not practice at all. Regarding students’ responsiveness, scores were intermediate, with about half the group rating the SBMT positively and the other half expressing reservations about its helpfulness. Both mindfulness practice and responsiveness were highly associated; students who practiced less also rated the SBMT negatively. Multilevel models indicated that student characteristics (sex, ethnicity, mental health risk profile), aspects of the school environment (school-level economic deprivation), and implementation factors (dose, quality of SBMT delivery) were predictors of mindfulness practice and/or responsiveness to the program.

It has been observed that universal SBMT requires students to use the skills taught if they are to derive any benefit.^12^^,^^39^ This study suggests that students’ self-reported mindfulness practice outside of lessons was low, with 70% to 90% of students practicing between “not at all to 3 times” each of the mindfulness practices. This finding is consistent with other studies that have recorded low rates of young peoples’ mindfulness practice.^14^ Although previous research has found no relationship in adolescents between out-of-school mindfulness practice and outcomes,^40^ mindfulness practice is usually considered essential in learning new mindfulness skills, which may explain the mixed results to date.^7^ Kuyken *et al.*^12^ found that students reporting more practice obtained greater improvements in stress, depression, and well-being, whereas Frank *et al.*^11^ found more beneficial effects on emotional awareness/regulation, mind wandering, impulse control, social connectedness, and reductions in substance use. Based on a conceptual model and previous research,^10^^,^^14^ we explored factors that could be potentially associated with students’ out-of-school mindfulness practice. The strongest effects were found for quality of delivery (ie, teacher competency, which depends on the teacher training and previous experience) and dose (ie, the number of SBMT sessions received). Both quality of delivery and dose are tractable predictors, and we hypothesize that optimizing teacher training and selection, as well as the number of sessions delivered, might increase subsequent out-of-school mindfulness practice, and in turn optimize student outcomes.

In line with previous research, students’ responsiveness to this SBMT curriculum was intermediate, with subgroups being either more or less responsive. We observed that female participants, Asian ethnicity, higher school-level economic deprivation, and higher SBMT dose and quality were associated with greater responsiveness. Some of these factors cannot be directly addressed by schools (eg, sex, ethnicity, school-level economic deprivation), but schools can implement SBMT curricula with careful attention to ensuring that they are adequately implemented/integrated into the prevailing school structure. For example, SBMT could fill an unmet need in schools with higher deprivation, whereas schools with less deprivation might already offer other programs to address student’s mental health. In the main trial, we found that higher risk of mental health problems was associated with worse outcomes,^30^ and here it was related to lower responsiveness. Schools might find it difficult to adapt a SBMT intervention to students with differing levels of mental health needs, by providing additional support for those with more risk, without triggering stigma. Moreover, it is possible that increasing young people’s awareness of problems without providing support could be counterproductive.^30^ It is a challenge for schools to provide universal SBMT alongside appropriate levels of support. However, findings emphasize the need for mindfulness instructors to be well trained (eg, trauma-informed, healing-centered training, etc) to deal with potential adverse responses, and secondary schoolteachers should be trained and collaborate with school counsellors and psychologists to offer extra support for students with greater needs.

Students’ experiences are useful in further interpreting these findings. Although most students chose not to respond to the open-ended questions, for those who did the most frequently reported experience was an increased awareness of feelings or bodily sensations. The “.b” curriculum asks students to pay attention to mind/body states and invites them to use a variety of mindfulness techniques. The second most frequently mentioned theme (managing feelings) may refer to some of these techniques. Consistent with previous qualitative research,^16^^,^^17^ we observed a mixed view of experiences, including both positive (optimism) and negative (rumination). In part, this could be due to the way in which we asked for this, using 2 open-ended questions, 1 question focused on positive experiences and 1 question on difficulties and challenges. It may explain the relatively high prominence of negative experiences compared with those in previous studies,^41^ but also sheds light on the low rates of practice and responsiveness, as some students expressed “no choice,” “couldn’t do it,” “didn’t do it,” “didn’t know,” “didn’t find it useful,” or found it “boring,” reported by 26% of students with an elaborated response (11% of those who answered).

Some students (10% of those with an elaborated response, 4% of those who answered) reported psychological “distress” associated with the SBMT. A previous study with students in late adolescence showed that difficult thoughts, emotions, and physical sensations were the most common sources of unpleasant experiences during MT, and that awareness of stress and unhappiness was experienced as somewhat harmful in 3% to 7% of participants.^42^ Perhaps, for students in this study, difficult thoughts and feelings were identified without having the appropriate levels of support to manage them. This is consistent with our finding that students with mental health problems were less responsive and had poorer outcomes.^30^ There are contrasting arguments for indicated vs universal SBMT. It has been established that around 3% to 10% of people will experience an adverse response to psychological interventions.^43^ Hence, a key issue here is the balance of benefit and cost, and the ethics of requiring students to participate in a universal course that may cause distress to some, together with the absence of clearly established mean improvements across the population. The main MYRIAD trial results,^9^ alongside our findings, point to the possible use of an indicated or targeted approach, rather than universal SBMT in early adolescence, in which students choose to participate in a curriculum. Nevertheless, the mindfulness curriculum that we tested was brief, which may have been insufficient to create positive change. Schools should consider programs that are longer and integrated into their school structure and culture to support youth and teacher well-being.^44^

It is also possible that older adolescents might practice and respond more to SBMT. The 14-to 18-year “window of opportunity”^45^ might be a key time for mindfulness to be implemented, due to heightened brain plasticity, self-reflection, social perspective taking, and a greater interest in understanding the self and others.^46^, ^47^, ^48^ More work is needed to explore possible age-related effects on mindfulness practice and responsiveness to SBMT.

The study has several limitations. This was a planned secondary analysis of a c-RCT. Thus, it is hypothesis generating, to inform future innovation and research. Our measures of practice and responsiveness were self-reported, which may not capture daily instances of practice and may be subject to bias (memory effects, social desirability, peer influence). Although we followed a theoretical model and previous research of potential predictors,^14^^,^^24^ observed relationships may be explained by other factors (macro-level policies, finances, standards, laws). Within this SBMT program, the extent of classroom-based mindfulness practice was limited, so generalization to the out-of-school environment was likely to be difficult, which could have led to the low rates of out-of-school practice, thereby threatening internal validity. Although our sample was generalizable to UK schools and students, further research is required in specialized schools and ethnic minorities. Our study suggests that students from different ethnic minorities may respond differently to SBMT. For example, among students identifying as of ethnicity other than White, specifically Asian ethnicity, there was some suggestion that they may show more responsiveness than other groups. Only a few studies have examined these aspects,^49^ and 1 study has suggested that SBMT could deter participation of minority ethnic groups.^50^ This requires further exploration and suggests the potential benefit of cultural adaptations in SBMT. Future research should be designed to specifically test the impact of these aspects on students’ mindfulness practice and responsiveness to SBMT. Finally, only a small proportion of students provided elaborated qualitative data. Using individual or group interviews would have allowed the top-level themes identified here to be explored in more depth, which needs further research.

The study has several strengths. It was based on a process evaluation embedded in a large c-RCT evaluating a universal SBMT. The external validity was maximized by a representative sample of students in secondary schools in the UK.^9^ Our design included triangulation across quantitative and qualitative data with both researchers’ framework and students’ experiences.^21^ This allowed making inferences with greater quality than those generated by each method separately. The content analyses of student’s experiences, although it came from only 2 open-ended questions, identified themes that help us to understand the trial results, and factors that might enhance future SBMT implementation. By providing the number and percentage of students who endorsed each theme, we would approximate how relevant they were to students. In sum, the integration of quantitative and qualitive approaches provides an answer to the question as to what students thought of this SBMT, both in terms of their self-reported practice and responsiveness, and their accounts of why they rated it as they did.^23^

Although the MYRIAD trial does not support universal SBMT using the “.b” curriculum,^9^ our findings suggest the need to innovate. We have seen that out-of-school mindfulness practice was low and responsiveness was intermediate, and that certain subgroups of students in particular contexts rated it differently. There is a question of whether we should move toward more indicated curricula for specific subgroups of students or schools and consider whether participation needs to be elective rather than compulsory. However, this approach is not without risks (eg, stigmatization). The influence of the quality of delivery seems critical, and training to deliver SBMT programs should incorporate how to cope with the difficulties raised by the young people, along with more experiential learning and supported practice. Co-designing SBMT programs with students and other key stakeholders with these considerations in mind might maximize accessibility, engagement, and effectiveness, by integrating ideas that enhance students’ investment and participation in the intervention. It would require highly trained and experienced teachers, and as well as attention to implementation facilitators and barriers at the student and school levels.
