# Supplementary material for: Do Adolescents Like School-Based Mindfulness Training? Predictors of Mindfulness Practice and Responsiveness in the MYRIAD Trial
Source: J Am Acad Child Adolesc Psychiatry. 2023 Nov;62(11):1256–69. doi: 10.1016/j.jaac.2023.02.016 (PMC10935541; doi:10.1016/j.jaac.2023.02.016)
Supplement: Supplemental Data [file mmc1.docx]

**Do adolescents like school-based mindfulness training? Predictors of mindfulness practice and responsiveness in the MYRIAD trial**

**Supplementary Materials**

**Supplement 1:** Implementation and delivery of the SBMT

**Supplement 2:** Details of the study measures

**Supplement 3:** Verification procedures regarding the qualitative work

**Table S1**: Selected characteristics of students included (responsiveness and practice) by follow-up status and cohort

**Figure S1:** Distribution of students’ mindfulness practice total scores

**Figure S2:** Distribution of students’ responsiveness total scores

**Table S2:** Distribution of home-based mindfulness practice and responsiveness to the SBMT across the ethnic groups specifying ethnic minorities

**Supplement 4:** Details of the concordance of judgements

**Table S3:** Codes and concordance indices

**Figure S3:** Word cloud representation of themes endorsement

**References**

**Supplement 1:** Implementation and delivery of the SBMT

The school-based mindfulness training (SBMT) was implemented in addition to/in place of Social Emotional Learning (SEL) in line with good practice guidance within the school curriculum. In the UK, delivering SEL is rarely mandatory, and therefore there is a wide variation across schools in terms of delivery. Nevertheless, SEL in the UK is usually taught as part of ‘*Personal, Social, Health and Economic Education*’ (PSHE) lessons. These lessons aim to enhance young people development as individuals and members of families and communities, trying to provide the knowledge, understanding, attitudes and practical skills to live healthily, safely, productively, and responsibly. PSHE provision is offered through a variety of methods, including regular scheduled lessons, drop-down days, within other subjects, and in tutor/form time (Department of Education, 2010). While SEL provision is not uniform, in general, it is intended to prepare students with the knowledge, skills, and attributes they need to manage their lives. It typically covers relationships, sex education, and physical and mental health education.

Recent UK Department of Education reports suggest that around 60% of secondary schools offer PSHE lessons that are at least ‘good’ (Department of Education, 2010). Determining whether schools have good PSHE provision is not an easy task. In the present study, for Cohort 1, schools were eligible for inclusion if their provision of PSHE met the following four criteria: (a) the presence of discrete, regular, named teaching time for PSHE; (b) a named PSHE lead; (c) a written PSHE policy; and (d) a named member of the senior leadership team responsible for PSHE. However, for Cohort 2, the ‘written PSHE policy’ criterion was modified to ‘documentation denoting clear strategic planning of SEL within the school’ (Montero-Marin et al., 2021). Experience in Cohort 1 indicated that schools do not always use the term ‘PSHE policy’ to denote strategic planning of SEL. Moreover, in some cases, there are schools that have an extensive, well-established, and well-documented SEL curriculum, indicative of a clear structure and strategy around SEL, but do not have this formalized as a school policy.

The SBMT was integrated into SEL teaching with good practice guidance in secondary schools, and it was aimed to teach mindfulness skills that support young people’s resilience. Mindfulness is a natural and trainable capacity to bring awareness to both inner (e.g., thoughts, feelings, body sensations) and outer experience (e.g., stressors, relationships), with qualities of curiosity and kindness (Feldman & Kuyken, 2019). All participating schools randomized to SBMT agreed to deliver the SBMT program to a minimum of three classes within years 8 and/or 9 or equivalent year groups across the UK nations (pupils aged 11-13). The SBMT program used was developed and adapted from mindfulness-based cognitive therapy (MBCT; Segal et al., 2013) over more than 5 years by three classroom teachers who were also experienced mindfulness practitioners. Their aim was to make it acceptable to young people across the full spectrum of functioning from mental health problems to flourishing, enabling all young people to use mindfulness skills to manage emotions, academic study, sport, sleep, and relationships. The SBMT program comprised several elements, delivered through the school curriculum, over several years, supported by teacher training. The SBMT program was taught to students in a set of 10 (30 to 50 minutes each) structured lessons taught in English in the spring terms (January-April), with support to continue use of mindfulness skills into the summer term. In the following school years, there were booster lessons intended to continue and support pupils’ further learning and ongoing mindfulness practice (e.g., lunchtime clubs or drop-in sessions). The SBMT program includes a combination of psychoeducation and practical skills involved in training the mind, learned in an experiential way, through short mindfulness practices which focus on the breath, body, and immediate experience. There is also classroom discussion of the application of new skills in everyday life. Its design aligns with principles identified as important for effectiveness in several reviews of schools-based programs that promote student mental health and well-being and teach social and emotional competence. These principles include: a) explicitly teaching skills and attitudes; b) tailoring components and approaches to the needs of young people; c) using a range of age-appropriate, interactive, experiential and lively teaching methods; d) providing age-appropriate resources that bring mindfulness to life (including a course booklet, a set of mindfulness exercises provided online, and mindfulness practices that are introduced through animations, and available as digital downloads); e) intensive, focused teacher education to build teachers’ self-efficacy and well-being; and f) program implementation which pays close attention to clarity and fidelity, supported by a manual and indicative script (Mindfulness in Schools Project, 2013).

The SBMT program includes strategies to support teachers in keeping mindfulness integral to the culture of their year group/the school as a whole. Examples of this include teacher catch-up days/support events, suggested schedules for progressive, regular mindfulness input throughout year groups, smartphone apps, and using parts of the SBMT program in core curriculum subjects. The study approach to implementing the SBMT was informed by theory and implementation science (Tudor et al., 2022), and was designed to be fully integrated into the school curriculum. All schools were supported with implementation guidance to increase the likelihood that it was introduced into the schools in ways that maintain its integrity and are sustainable. Implementation started with engaging the school leadership team, and then identifying a potential pool of teachers from within the school who could be trained and timetabled to deliver it to the pupils. The selected teachers then went through a training program (i.e., Mindfulness-Based Cognitive Therapy for Life, MBCT-L (Strauss et al., 2021)) followed by a 4-day training course in how to deliver SBMT to students. Teachers received mentoring from qualified teachers who had also been trained in and delivered both the MBCT-L and SBMT. They a) had taught at least five MBCT-L classes since their qualification; b) had a extensive experience of working with secondary school teachers in school settings; c) were trained in by the developers of the SBMT curriculum to deliver the training; d) were registered on the UK listing of mindfulness trainers; and d) adhered to the Good Practice guidelines for mindfulness instructors (http://mindfulnessteachersuk.org.uk/#guidelines). After the training course, mentors went into each school for a few days, supervised a lesson per teacher and gave face-to-face feedback, met with all the teachers in each school for a discussion and summary of the visit, and performed a collective practice reinforcing self-confidence, reassurance, guiding practices and inquiry skills. After the visit, mentors shared all written feedback of their observed lesson and were available by phone for any support or query at the teachers’ convenience.

**Supplement 2:** Details of the study measures

*Mindfulness practice*

We assessed the frequency of students’ home-based mindfulness practice at post-intervention using a 6-item measure specifically designed for the purpose of the study. Questions were answered on a 6-point Likert-type scale as follows: 0 = ‘Never’, 1 = ‘Once’, 2 = ‘Once a week’, 3 = ‘2/3 times a week’, 4 = ‘Several times a week’, 5 = ‘Everyday’. The items that compose this scale are the following: ‘During the course you were taught a range of mindfulness practices. How often did you practice being mindful?’, ‘During the course you were invited to pause and focus on your breathing by doing a 7-11 or FOFBOC or a ‘.b’ (i.e., stop, breathe and be). How often did you do this?’, ‘During the course you were taught to use ‘beditation’ as a way of helping you get to sleep. How often did you do this?’, ‘During the course you were asked to be mindful in your everyday lives, for example walk a short distance mindfully, or eat a mouthful of food mindfully. How often did you do this?’, ‘During the course you were asked to notice stress in your body, e.g., ‘stress signature’ in difficult times, noticing where in the body you were feeling stress. How often did you do this?’, and ‘During the course you were taught to think about your thoughts as passing objects such as buses, clouds or rivers that pass through your mind. How often did you do this?’. All responses were summed and divided by the number of items (range: 0-5; with higher scores representing a higher frequency of students’ mindfulness practice). The internal consistency (Cronbach’s alpha (α)) of the mindfulness practice scale was α = 0.89.

*Responsiveness*

We assessed student responsiveness to SBMT at post-intervention using a 5-item measure that was adapted from a previous study (Bluth et al., 2016). Each question was answered on a 11-point Likert-type scale (with anchors at 0 = ‘Not at all’, and 10 = ‘A great deal’, with no intermediate labels for the values in between). The items that compose this scale are the following: ‘How much does what’s being taught in these lessons make sense to you in helping you to deal with issues young people face?’, ‘Do you think that these lessons will help you have a healthier lifestyle?’, ‘Would you recommend these lessons to a friend?’, ‘How important do you think it is that these lessons are available to young people?’, ‘How successful do you believe these lessons are likely to be in decreasing problems or issues that young people have?’. Total scores were calculated by summing the responses divided by the number of items (range: 0-10; higher scores represented higher responsiveness). Internal consistency was α = 0.95.

*Predictors of* *students’ mindfulness practice and responsiveness*

**Student** factors included: age (11-13 years at entry to the trial), self-identified gender (‘male’, ‘female’), ethnicity (‘White’, ‘Arab’, ‘Asian’, ‘Black/African/Caribbean’, ‘Mixed/Multiple ethnic groups’, ‘Others’; as it is shown in the study results, the largest proportion of students identified as ‘White’, with a lower proportional representation of other ethnic subgroups, which is consistent with the broader UK student population. We acknowledge that a detailed exploration of the mindfulness practice and responsiveness of other ethnic groups awaits further research, with studies being designed to address this research question), and risk for mental health problems (‘at risk’, ‘not at risk’; derived by carrying out a latent profile analysis (LPA) that included the baseline characteristics: age, gender, ethnicity, risk for depression, social-emotional-behavioral difficulties, well-being, as well as school urbanicity, school-level economic deprivation, and SEL ethos —which refers to the values that the school represents in relation to the way staff and students relate, the development of bonds, and the opportunities for participation in positive social activities (Weare & Nind, 2011)).

The risk for mental health was assessed by developing a series of LPAs that were conducted using maximum likelihood (ML) estimation with cluster (students within schools) robust standard errors. For that, LPA models containing one to eight latent profiles in a randomly selected sub-sample were carried out. To validate the structure of the selected latent profile model, we tested LPA models in the other half of the sample, and all subsequent analyses were then developed with the total sample. For model selection, we used the Akaike information criterion (AIC), consistent Akaike information criterion (CAIC), Bayesian information criterion (BIC), sample-size-adjusted BIC (sBIC), Lo–Mendell–Rubin adjusted likelihood ratio test (LMR-LRT), bootstrapped likelihood ratio test (BLRT), index of classification accuracy (Entropy), Elbow Plots, model parsimony, and the theoretical interpretability (Nylund et al., 2007). Considering all this, a two-profile model was selected. Students were assigned into their most likely baseline profile based on BCH weights, reflecting the measurement error of the latent profile variable. The largest subgroup of students (72.8%) was characterized by lower values of risk for depression and social-emotional-behavioral difficulties, as well as higher values of well-being. This subgroup was also younger, more often identified as males, and other ethnic backgrounds than whites, had a higher SEL ethos, and were more often from rural areas. Students in this subgroup were much less likely to be at risk of suffering from mental health problems, and thus, this sub-group was labelled as ‘low risk’. On the contrary, the other subgroup of students (27.2%) had higher values of risk for depression and social-emotional-behavioral difficulties, as well as lower values of well- being, and were older, more often identified as females and ‘whites’, had a lower SEL ethos, and were more often from urban areas. Students in this subgroup were more likely to be at risk of suffering from mental health problems, and thus, this subgroup was labelled as ‘high risk’. More details about the LPAs and the characteristics of the latent profiles can be found in Montero-Marin et al. (2022).

**Teacher** variables included: years of teaching experience at baseline, gender (‘male’, ‘female’), ethnicity (‘white’, ‘other ethnic group’), and burnout at baseline. Burnout was assessed with the Maslach Burnout Inventory-Educators Survey (MBI-ES; Maslach et al., 1996). The ‘Maslach Burnout Inventory Educators Survey’ is a questionnaire that was specifically designed for teachers working in an educational setting. This questionnaire determines how teachers view their job and their reactions to their work pertaining to how often they experience various aspects of occupational burnout, including feelings about students, work, and success. There are 22 items that form 3 scales: Emotional Exhaustion (e.g., ‘I feel emotionally drained from my work’), Depersonalization (e.g., ‘I feel I treat some students as if they were impersonal objects’), and (lack of) Personal Accomplishment (e.g., ‘I deal very effectively with the problems of my students’, item reversed). Participants respond to a 7-point Likert-type scale according to the frequency the individual identifies with each statement (from 0, ‘Never’, to 6, ‘Every day’). Mean scores are used to calculate each scale. We reversed the Personal Accomplishment items so that higher scores in the three scales indicate higher presence of burnout symptoms. We used the mean of all items to calculate the MBI total score (range: 0-132). This scale has been validated for use among teaching populations (Byrne, 1993; Kokkinos, 2006). Internal consistency of the MBI total score in our study at baseline was α = 0.88.

**School** variables were largely obtained from governmental online resources (Ford et al., 2021), and included urbanicity (urban, rural) as a school context factor, the proportion of students within the school eligible for free school meals at baseline (i.e., school-level economic deprivation) as a school community factor, and student-to-teacher ratio and school SEL ethos (using an index that ranges between 0 and 100) at baseline, as operational features of the school.

The school SEL ethos refers to the underlying values and attitudes that the school represents in relation to the way staff and students relate, as well as the development of bonds between youth and adults, and the opportunities for participation in positive social activities (Weare & Nind, 2011). We developed a school SEL ethos measure gathering existing data from relevant sources at baseline identifying those variables that map onto the hypothesized construct. For that, the following school-level measures were considered: a) *Personal, Social, Health and Economic Education* (PSHE) *provision*, based on 16 quality indicators (schools were assigned a score, out of 16) that were identified through a review via expert consultation (Department of Education, 2010), and created specifically for this trial (Montero-Marin et al., 2022); b) S*chool ecology*, as an aggregated measure from averaged teacher ratings based on the teacher version of the ‘School Climate and Connectedness Survey’ (Association of Alaska School Boards, 2015), that included the domains of ‘school leadership and involvement’, ‘staff attitudes’, and ‘respectful climate’, with a Cronbach’s alpha value for the total score (which ranges from 1 to 5) of α = 0.90; c) *Ofsted school quality rating*, operationalized by the official school quality rating at baseline (https://www.gov.uk) as ‘outstanding’ (1), ‘good’ (2), ‘requires improvement’ (3), and ‘inadequate’ (4); and d) an *independent researcher rating of the school’s commitment to SEL* based on the direct observation of the school using the quality indicators referred above (range: from 0 to 16). All the measures were re-scaled to a new range, from 0 to 4, to ensure that all the variables contributed equally to the computation of the final index. Optimal implementation of parallel analysis, with 500 random correlation matrices, was used as a dimensionality test to decide on the number of dimensions. The advised number of dimensions was 1, which explained a 65% of real-data variance. The robust unweighted least squares method was employed for factor extraction, which produced loadings between 0.54 and 0.67, and an internal consistency of α = 0.72. Factor scores were calculated by means of Bayes Expected a Posteriori (EAP) estimates transformed to T-scores (range: 0-100), with higher scores representing a more conducive school SEL ethos towards the promotion of social, emotional, and mental well-being. Further details about the SEL ethos measure that we used in the present study can be found in Montero-Marin et al. (2022).

**Implementation** factors included pupil, class, and school levels and comprised the following:

*Dose* received (the number of ‘.b’ SBMT sessions that students attended, which ranged between 0 and 10, and that was completed by the teacher teaching the lessons).

*Fidelity* to the original ‘.b’ program, measured as the percentage of the standardized curriculum that was covered overall in two randomly selected lessons per intervention class. All SBMT lessons were filmed and a randomly chosen subset of 2 out of the 10 possible lessons from each class were evaluated. For each selected lesson, independent evaluators who were experienced mindfulness instructors, indicated whether key curriculum elements were delivered or not. The ‘MiSP .b Fidelity Checklist’ (https://mindfulnessinschools.org) was used by the two independent evaluators. These ratings were summarized as the percentage of curriculum elements covered per lesson, and they were averaged across the two ‘.b’ randomly selected lessons to provide a percentage of elements covered per intervention class.

*Quality* of SBMT delivery was considered as the teaching competency, and it was rated using the ‘Mindfulness-Based Interventions–Teaching Assessment Criteria’ (MBI-TAC; Crane et al., 2012; Crane et al., 2013; Crane et al., 2020). All SBMT lessons were filmed and a randomly selected subset of 2 out of the 10 possible lessons from each class were rated. Lessons were rated by one of four different assessors using an adapted version of the MBI-TAC for the teaching context (MBI-TAC-Teach, see https://mbitac.bangor.ac.uk/documents/MBI-TAC-for-schools.pdf). External evaluators were MBI teachers, who had a recognized mindfulness training pathway, were qualified to teach the SBMT (‘.b’) with more than two years of experience, and were also qualified classroom teachers. They were trained in the use of the MBI-TAC-Teach assessment (an MBI-TAC version to be used to rate classroom teachers, teaching mindfulness to young people in school contexts) by taking part in two days of training where the MBI-TAC-Teach was introduced by two experienced supervisors. They all had experience of being rated by the MBI-TAC in their own teaching pathway so all of them were familiar with this tool. The training focused on the aspects of the MBI-TAC-Teach tool and was based on collective discussions and evaluations of some case studies to ensure standardization. Evaluators were allowed time to rate some examples independently to ensure consistency and that these ratings were within an acceptable range for all the evaluators at the end of the training. All evaluators took part in regular supervision sessions with the aim of ensuring/maximizing assessment standardization. Competence is rated across 6 domains on a 6- point scale (1 = ‘incompetent’, 2 = ‘beginner’, 3 = ‘advanced beginner’, 4 = ‘competent’, 5 = ‘proficient’, and 6 = ‘advanced’). The domains assess: coverage, pacing and organization of session curriculum; relational skills; embodiment of mindfulness; guiding mindfulness practices; conveying course themes through interactive enquiry and didactic teaching; and holding the group learning environment. Evaluators provided competency ratings on the 6 domains, and an overall competency rating per lesson (based on their own overall assessment rather than a sum score of the 6 domains), for the two randomly selected lessons per intervention class. Based on the two lessons, an overall rating per domain for that class was completed, and then used by evaluators to provide one overall final competency rating per class as a measure of the quality of the intervention delivery.

*Reach*, which refers to the rate of involvement of program participants, and it is mainly concerned with questions relating to the percentage of the eligible population who took part in the intervention (Durlak, & DuPre, 2008). In the present study, student reach was considered at post-intervention as the proportion of students attending more than 67% (Panayiotou et al., 2020) of ‘.b’ SBMT lessons relative to the study’s year group school population.

*SEL delivery (i.e., additions to/replacement of PSHE curriculum):* it was measured to what extent the SBMT program was: a) additive to the PSHE curriculum, so that it was unaffected (either ‘.b’ did not take place in PSHE lessons, or if they did, PSHE curriculum content was either not removed, or taught elsewhere if removed); b) partially additive/partially substitutive, and thus PSHE curriculum content was condensed to make room for ‘.b’ content; c) substitutive, and therefore PSHE content was removed to make room for ‘.b’ and it was not taught elsewhere. To assess the integration of the SBMT program into the SEL school curriculum, we surveyed all schools and crosschecked their responses with student attendance registers.

Further details on the implementation measures can be found in Montero-Marin et al. (2022).

*Open-ended questions*

We asked about pupils’ experiences of SBMT at post-intervention using two open-ended questions: one focused on positive experiences (‘Did you notice any thoughts, feelings, or sensations during or after the ‘.b’ lessons that were positive or interesting to you, or made you think or feel differently about things?’), and one on difficulties/challenges (‘Did you notice any thoughts, feelings, or sensations during or after the ‘.b’ lessons that were difficult or challenging for you?’), to capture the complete range of possible experiences.

**Supplement 3:** Verification procedures regarding the qualitative work

We ensured *authenticity* (Holloway & Wheeler, 2002) by including an opportunity for students to opt out, and then for those who opted to respond, an opportunity to be an active participant. This took several forms: a) describing their experiences through a narrative approach with minimally structured questions, and b) running a range of public engagement activities, such as ‘The Young Researcher Challenge’ ([The MYRIAD Public Engagement Programme, 2022](https://myriadproject.org/engagement-activities/the-myriad-public-engagement-programme/)). *Verification* procedures included using multiple researchers in the analysis to compare interpretations of the data (i.e., investigator triangulation), as well as triangulation between quantitative and qualitative methods (i.e., method triangulation). *Dependability* was maximized making explicit the analysts’ narratives by taking reflexive notes throughout the analytical phase that clarified making decision processes (Lincoln & Guba, 1985). These researcher’s notes and interpretations, together with the original verbatim transcripts, were shared within the extended research team for discussion to establish *credibility* (Holloway & Freshwater, 2007). We present detailed evidence in the form of quotations in the present report. *Transferability* within similar contexts was supported by receiving feedback from teachers and students at the stages of the interim and final findings (Lincoln & Guba, 1985). Therefore, our results were *applicable* (Agius, 2013) to the type of setting (UK secondary schools) and people (early adolescents) object of study, to whom the emergent themes apply based on the identification and circumscription of similar settings and participants in the study (see the example of notes from two students’ feedback in the next two pages below). *Plausibility* was derived from achieving accuracy in the data collection process, using two open-ended questions that created a context in which both positive and negative experiences with SBMT could be described anonymously (Roller & Lavrakas, 2015). The systematic collection of materials and documentation permits an independent auditor to come to conclusions about the data, allowing a test of *confirmability*. Qualitative analyses were performed using MAXQDA v2020.

**Example of notes from two students’ feedback**

Physical Sensations

AD - I remember the one where you had to shut your eyes and concentrate on parts of my body.

JD – didn’t feel any physical sensations.

Managing Feelings

JD – sometimes the breathing worked.

AD – I didn’t help me; I didn’t have many problems at that time.

Changes in energy

JD – it made me quite drowsy

AD – it made me feel a bit tired, I’d agree.

Changes in focus

AD – my focus stayed the same.

JD – my focus stayed the same.

Distress

JD – Year 8 was quite a peaceful year; I felt no distress.

AD – I remember the bus one, the students in my lesson made me feel comfortable anyway, perhaps it would have been different in a room of strangers.

Boredom

JD – My class was art and I felt it was a relaxing lesson anyway, but I felt I was missing out on art by doing it.

AD – I wasn’t bored as I liked my class and got on with them all, so we made it fun.

Rumination

JD – I said on some days I felt quite agitated as it was getting repetitive, but that was the only reason.

AD – nothing came back to me.

Can’t do it

JD – I only did the breathing ones, I didn’t do anything else after school.

AD – I can’t remember.

I don’t know

JD - Didn’t have an impact.

AD – I think it might work in time, but I don’t practice.

Optimism

JD – at the time, yes, but over time I got more stressed with school, so it was short-lived.

AD – nothing really changed.

Not useful

JD – I enjoyed the sleeping one.

AD – 50/50

Curiosity

JD – I was not curious

AD – I was not curious

Appreciation

JD – it increased my gratitude for things, and appreciation.

AD – it didn’t affect this for me.

Didn’t do it

Neither of us did it after the lessons.

Self Confidence

JD – not really

AD – didn’t make a difference

Attentive to others

JD – 50/50

AD – didn’t make a difference

Better sleep

No difference

No choice

JD – I agree that our views

AD – I felt this over time, that I was being expected to engage with something that was no longer working.

**Table S1** Selected characteristics of students included (practice/responsiveness) by follow-up status and cohort

| **Pupil characteristics** |  | **Students lost at post-intervention*** | | | | |  | **Remaining students**** | | | | |
| --- | --- | --- | --- | --- | --- | --- | --- | --- | --- | --- | --- | --- |
|  |  | **Cohort 1** |  | **Cohort 2** |  | **Total** |  | **Cohort 1** |  | **Cohort 2** |  | **Total** |
|  |  | **N = 96** |  | **N = 541** |  | **N = 637** |  | **N = 455** |  | **N = 3140** |  | **N = 3595** |
|  |  |  |  |  |  |  |  |  |  |  |  |  |
| Age^†^ – M (SD) |  | 11.6 (0.7) |  | 11.7 (0.6) |  | 11.7 (0.6) |  | 11.6 (0.6) |  | 11.7 (0.6) |  | 11.7 (0.6) |
| Gender^††^ – Female, n (%) |  | 50 (52.1) |  | 213 (39.4) |  | 263 (41.3) |  | 282 (62.0) |  | 1805 (57.5) |  | 2087 (58.1) |
| Ethnicity^†††^ – White, n (%) |  | 80 (83.3) |  | 391 (72.3) |  | 471 (73.9) |  | 380 (83.5) |  | 2386 (76.0) |  | 2766 (76.9) |
| Risk for mental health^††††^ – Yes, n (%) |  | 37 (38.5) |  | 191 (35.3) |  | 228 (35.8) |  | 125 (27.5) |  | 823 (26.2) |  | 948 (26.4) |
| *Defined as those pupils with missing data on overall responsiveness l score. **Defined as those pupils with overall responsiveness score at post-intervention.  ^†^ Sample size in lost to follow-up group: 637: Cohort 1: 96; Cohort 2: 541. Sample size in remaining students’ group: 3595: Cohort 1: 455; Cohort 2: 3140.  ^††^ Sample size in lost to follow-up group: 614: Cohort 1: 95; Cohort 2: 519. Sample size in remaining students’ group: 3543: Cohort 1: 455; Cohort 2: 3088.  ^†††^ Sample size in lost to follow-up group: 612: Cohort 1: 95; Cohort 2: 517. Sample size in remaining students’ group: 3533: Cohort 1: 454; Cohort 2: 3079.  ^††††^ Sample size in lost to follow-up group: 637: Cohort 1: 96; Cohort 2: 541. Sample size in remaining students’ group: 3595: Cohort 1: 455; Cohort 2: 3140. | | | | | | | | | | | | |
| **Pupil characteristics** |  | **Students lost at post-intervention*** | | | | |  | **Remaining students**** | | | | |
|  |  | **Cohort 1** |  | **Cohort 2** |  | **Total** |  | **Cohort 1** |  | **Cohort 2** |  | **Total** |
|  |  | **N = 89** |  | **N = 506** |  | **N = 595** |  | **N = 462** |  | **N = 3175** |  | **N = 3637** |
|  |  |  |  |  |  |  |  |  |  |  |  |  |
| Age^†^ – M (SD) |  | 11.6 (0.7) |  | 11.7 (0.6) |  | 11.7 (0.6) |  | 11.7 (0.6) |  | 11.7 (0.6) |  | 11.7 (0.6) |
| Gender^††^ – Female, n (%) |  | 46 (51.7) |  | 200 (39.5) |  | 246 (41.3) |  | 286 (61.9) |  | 1818 (57.3) |  | 2104 (57.8) |
| Ethnicity^†††^ – White, n (%) |  | 73 (82.0) |  | 369 (72.9) |  | 442 (74.3) |  | 387 (83.8) |  | 2408 (75.8) |  | 2795 (76.8) |
| Risk for mental health^††††^ – Yes, n (%) |  | 35 (39.3) |  | 179 (35.4) |  | 214 (36.0) |  | 127 (27.5) |  | 835 (26.3) |  | 962 (26.5) |
| *Defined as those pupils with missing data on overall mindfulness practice score. **Defined as those pupils with overall practice score at post-intervention.  ^†^ Sample size in lost to follow-up group: 595: Cohort 1: 89; Cohort 2: 506. Sample size in remaining students’ group: 3637: Cohort 1: 462; Cohort 2: 3175.  ^††^ Sample size in lost to follow-up group: 575: Cohort 1: 88; Cohort 2: 487. Sample size in remaining students’ group: 3582: Cohort 1: 462; Cohort 2: 3120.  ^†††^ Sample size in lost to follow-up group: 573: Cohort 1: 88; Cohort 2: 485. Sample size in remaining students’ group: 3572: Cohort 1: 461; Cohort 2: 3111.  ^††††^ Sample size in lost to follow-up group: 595: Cohort 1: 89; Cohort 2: 506. Sample size in remaining students’ group: 3637: Cohort 1: 462; Cohort 2: 3175. | | | | | | | | | | | | |

**Figure S1:** Distribution of students’ mindfulness practice total scores

| **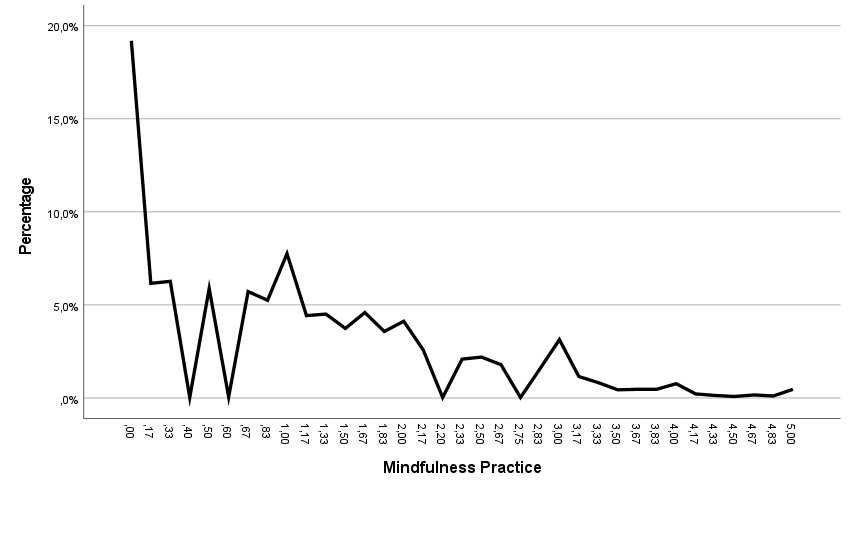** |
| --- |

Note: ‘0’ = Never, ‘1’ = Once, ‘2’ = Once a week, ‘3’ = 2/3 times a week, ‘4’ = Several times a week, ‘5’ = Everyday

**Figure S2:** Distribution of students’ responsiveness total scores

| **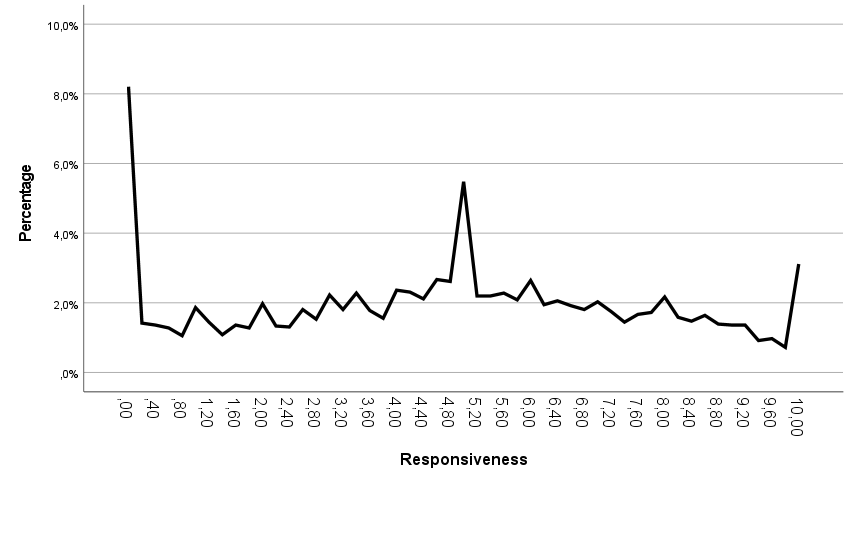** |
| --- |

Note: ‘0’ = Not at all, ‘10’ = A great deal

**Table S2:** Distribution of home-based mindfulness practice, and responsiveness to the SBMT across the ethnic groups specifying ethnic minorities

**Table S2**

| **Mindfulness practice total score** | | | |
| --- | --- | --- | --- |
| **Ethnic groups** | **N** | **M** | **SD** |
| White | 2779 | 1.15 | 1.06 |
| Arab | 63 | 0.92 | 1.05 |
| Asian | 314 | 1.28 | 1.11 |
| Black/African/Caribbean | 159 | 1.14 | 1.13 |
| Mixed/Multiple Ethnic Groups | 158 | 1.04 | 1.02 |
| Other Ethnic Group | 78 | 1.45 | 1.12 |
| Note: ‘0’ = Never, ‘1’ = Once, ‘2’ = Once a week, ‘3’ = 2/3 times a week, ‘4’ = Several times a week, ‘5’ = Everyday | | | |
| **Responsiveness total score** | | | |
| **Ethnic groups** | **N** | **M** | **SD** |
| White | 2766 | 4.66 | 2.89 |
| Arab | 64 | 4.38 | 2.83 |
| Asian | 311 | 5.30 | 2.85 |
| Black/African/Caribbean | 156 | 4.84 | 2.80 |
| Mixed/Multiple Ethnic Groups | 157 | 4.55 | 2.67 |
| Other Ethnic Group | 79 | 5.19 | 2.85 |

Note: ‘0’ = Not at all, ‘10’ = A great deal

**Supplement 4:** Details of the concordance of judgements

We determined the adequacy of content analysis coding precision by two researchers independently coding 10% of the entries and calculating the Cohen’s kappa coefficient (κ) to estimate levels of agreement. Cohen’s kappa quantifies the degree of agreement between the two observers in terms of the presence or absence of the feature, after correcting for chance agreement (Cohen & Jacob, 1960). We calculated the standard error (SE) associated with this coefficient as well as its confidence interval at 95% (95% CI). We also contrasted the kappa value to the behaviour of the random classifier (Efron & Tibshirani, 1993). In general, a kappa value >0.40 is considered acceptable, and >0.75 is believed to be excellent (Altman, 1991; Landis & Koch, 1977).

The global kappa value was 0.97 (95% CI = 0.96 to 0.98). Table *a* shows the concordance of judgments according to the emerging classification system for the final list of theme categories and a randomly selected 10% of the entries. The obtained kappa coefficients were appropriate in all the codes, with values between 0.44 (physical sensations) and 0.99 (no choice). We also calculated the percentage of agreement among the two referees. The general agreement among the two referees was 98.2%.

*Table S3: Codes and concordance indices*

| **Codes** | **%** | **κ** | **95% CI** |
| --- | --- | --- | --- |
| Distress | 93.9 | 0.45 | 0.26 to 0.61 |
| Rumination | 99.3 | 0.88 | 0.74 to 0.99 |
| Can’t do it | 99.3 | 0.66 | 0.31 to 0.99 |
| Boredom | 99.1 | 0.85 | 0.71 to 0.99 |
| No choice | 99.9 | 0.99 | 0.99 to 0.99 |
| Not useful | 93.4 | 0.46 | 0.29 to 0.62 |
| Didn’t do it | 99.9 | 0.99 | 0.99 to 0.99 |
| Physical sensations | 93.0 | 0.44 | 0.27 to 0.60 |
| Curiosity | 99.8 | 0.80 | 0.41 to 0.99 |
| New perspective | 97.7 | 0.68 | 0.49 to 0.87 |
| Managing feelings | 95.6 | 0.81 | 0.72 to 0.89 |
| Optimism | 99.1 | 0.77 | 0.56 to 0.99 |
| Self-confidence | 97.8 | 0.96 | 0.58 to 0.99 |
| Appreciation | 99.5 | 0.67 | 0.23 to 0.99 |
| Attentive to others | 99.3 | 0.57 | 0.13 to 0.99 |
| Energy | 99.5 | 0.93 | 0.82 to 0.99 |
| Focus | 99.1 | 0.88 | 0.77 to 0.99 |
| Better sleep | 99.8 | 0.98 | 0.97 to 0.99 |
| I don’t know | 99.8 | 0.91 | 0.73 to 0.99 |
| **Total** | 98.2 | 0.97 | 0.96 to 0.98 |

% = percentage of agreement between the 2 referees; κ = kappa (2 referees and 2 classification categories); 95% CI = confidence interval at 95%.

**Figure S3**. Word cloud representation of themes endorsement


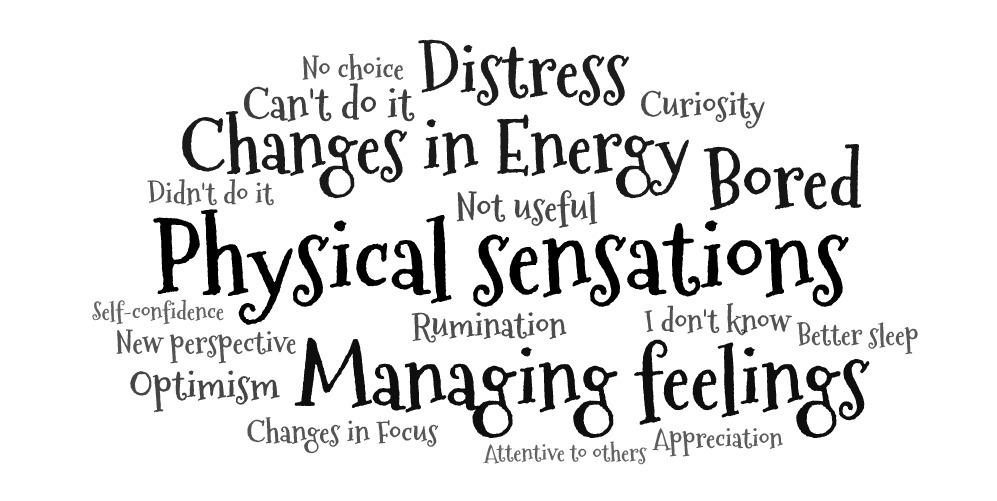


Note: A word cloud is a text-based data visualization format in which the bigger and bolder the word appears, the more frequently it has been endorsed. Definitions of themes, examples of verbatim, frequencies and percentages can be seen in the Table 5 of the main manuscript document.

**References for the Supplements**

Agius, S. (2013). Qualitative research: Its value and applicability. The Psychiatrist. 2013;37(6):204-206. https://doi.org/10.1192/pb.bp.113.042770

Altman, D.G. Practical statistics for medical research. New York: Chapman and Hall; 1991.

Association of Alaska School Boards. School climate and connectedness survey [measurement instrument]. Juneau: Association of Alaska School Boards; 2015.

Bluth K., Campo R. A., Pruteanu-Malinici S., Reams A., Mullarkey M., Broderick P. C. A school-based mindfulness pilot study for ethnically diverse at-risk adolescents. Mindfulness. 2016;7(1):90-104. https://doi.org/10.1007/s12671-014-0376-1

Byrne, B. M. The Maslach Burnout Inventory: Testing for factorial validity and invariance across elementary, intermediate, and secondary teachers. Journal of Occupational and Organizational Psychology 1993;66:197-212.

Cohen, Jacob (1960). "A coefficient of agreement for nominal scales". Educational and Psychological Measurement 20 (1):37-46. https://doi.org/10.1177/001316446002000104

Crane, R. S., Koerbel, L., Sansom, S., et al. Assessing mindfulness-based teaching competence: good practice guidance. Global advances in Health and Medicine. 2020;9. https://doi.org/10.1177/2164956120973627.

Crane, R. S., Eames, C., Kuyken, W., et al. Development and validation of the Mindfulness-Based Interventions - Teaching Assessment Criteria (MBI:TAC). Assessment. 2013;20:681-8 https://doi.org/10.1177/1073191113490790.

Crane, R. S., Kuyken, W., Williams, J. M. G. Competence in teaching mindfulness- based courses: Concepts, development and assessment. Mindfulness. 2012;3:8. https://doi.org/10.1007/s12671-011-0073-2

Department of Education. Personal, social, health and economic (PSHE) education: A mapping study of the prevalent models of delivery and their effectiveness. London; 2010.

Durlak, J. A., DuPre, E. P. Implementation matters: a review of research on the influence of implementation on program outcomes and the factors affecting implementation. American journal of community psychology. 2008;41(3-4):327-50. https://doi.org/10.1007/s10464-008-9165-0

Efron B, Tibshirani RJ. An introduction to the bootstrap. New York: Chapman and Hall; 1993.

Feldman, C., & Kuyken, W. (2019). Mindfulness: Ancient wisdom meets modern psychology. The Guilford Press.

Ford, T., Degli Esposti, M., Crane, C., Taylor, L., Montero-Marín, J., Blakemore, S. J., Bowes, L., Byford, S., Dalgleish, T., Greenberg, M. T., Nuthall, E., Phillips, A., Raja, A., Ukoumunne, O. C., Viner, R. M., Williams, J., Allwood, M., Aukland, L., Casey, T., De Wilde, K., … Kuyken, W. (2021). The Role of Schools in Early Adolescents' Mental Health: Findings From the MYRIAD Study. Journal of the American Academy of Child and Adolescent Psychiatry, 60(12), 1467–1478. https://doi.org/10.1016/j.jaac.2021.02.016

Holloway, I., Freshwater, H. Trustworthiness and Authenticity in Narrative Research. Critical Issues in Narrative Research; Willey; 2007, pp. 103-116. https://doi.org/10.1002/9781444316513.ch10

Holloway, I., Wheeler, S. Qualitative research in nursing. Oxford: Blackwell Science; 2002.

Kokkinos, CM. Factor Structure and Psychometric Properties of the Maslach Burnout Inventory- Educators Survey among Elementary and Secondary School Teachers in Cyprus. Stress and Health 2006;22,25-33.

Landis, J. R., Koch, G. G. The measurement of observer agreement for categorical data. Biom. 1977;13:159-174. https://doi.org/10.2307/2529310

Lincoln, Y. S., Guba, E. G. Naturalistic Inquiry. Beverly Hills, CA: Sage Publications, Inc; 1985.

Maslach C, Jackson SE, Leiter MP. Maslach burnout inventory manual, third edition. Palo Alto, California: Press CP 1996.

Montero-Marin, J., Allwood, M., Ball, S., Crane, C., De Wilde, K., Hinze, V., Jones, B., Lord, L., Nuthall, E., Raja, A., Taylor, L., Tudor, K., MYRIAD Team, Blakemore, S. J., Byford, S., Dalgleish, T., Ford, T., Greenberg, M. T., Ukoumunne, O. C., Williams, J., … Kuyken, W. School-based mindfulness training in early adolescence: what works, for whom and how in the MYRIAD trial? Evidence-based mental health. 2022;25(3):117-24. <https://doi.org/10.1136/ebmental-2022-300439>

Montero-Marin, J., Nuthall, E., Byford, S., Crane, C., Dalgleish, T., Ford, T., Ganguli, P., Greenberg, M. T., Ukoumunne, O. C., Viner, R. M., Williams, J., MYRIAD team, & Kuyken, W. (2021). Update to the effectiveness and cost-effectiveness of a mindfulness training programme in schools compared with normal school provision (MYRIAD): study protocol for a randomised controlled trial. Trials, 22(1), 254. https://doi.org/10.1186/s13063-021-05213-9

Nylund KL, Asparouhov T, Muthén BO. Deciding on the number of classes in latent class analysis and growth mixture modeling: a monte carlo simulation study. Structural Equation Modeling: A Multidisciplinary Journal. 2007;14:535-69. <https://doi.org/10.1080/10705510701575396>

Panayiotou, M., Humphrey, N., Hennessey, A. Implementation matters: Using complier average causal effect estimation to determine the impact of the Promoting Alternative Thinking Strategies (PATHS) curriculum on children’s quality of life. Journal of Educational Psychology. 2020;112(2):236-53. https://doi.org/10.1037/edu0000360

Roller, M. R., Lavrakas, P. J. Applied Qualitative Research Design: A Total Quality Framework Approach. 2015. New York: Guilford Press.

The MYRIAD Public Engagement Programme. Accessed 15 July 2022. https://myriadproject.org/engagement-activities/the-myriad-public-engagement-programme/

Weare, K., Nind, M. Mental health promotion and problem prevention in schools: what does the evidence say? Health Promotion International. 2010;25:29-69 <https://doi.org/10.1093/heapro/dar075>

Segal ZV, Williams JMG, Teasdale JD. Mindfulness-Based Cognitive Therapy For Depression. New York: Guildford Press 2013.

Mindfulness in Schools Project. (2016). How to teach .b. Teachers Notes. Curriculum available from: http://mindfulnessinschools.org/.

Tudor, K., Maloney, S., Raja, A., Baer, R., Blakemore, S. J., Byford, S., Crane, C., Dalgleish, T., De Wilde, K., Ford, T., Greenberg, M., Hinze, V., Lord, L., Radley, L., Opaleye, E. S., Taylor, L., Ukoumunne, O. C., Viner, R., MYRIAD Team, Kuyken, W., … Montero-Marin, J. (2022). Universal Mindfulness Training in Schools for Adolescents: a Scoping Review and Conceptual Model of Moderators, Mediators, and Implementation Factors. Prevention science : the official journal of the Society for Prevention Research, 23(6), 934–953. https://doi.org/10.1007/s11121-022-01361-9

Strauss, C., Gu, J., Montero-Marin, J., Whittington, A., Chapman, C., & Kuyken, W. (2021). Reducing stress and promoting well-being in healthcare workers using mindfulness-based cognitive therapy for life. International journal of clinical and health psychology: IJCHP, 21(2), 100227. https://doi.org/10.1016/j.ijchp.2021.100227
